# Identification of a multi-modal mechanism for Se(VI) reduction and Se(0) allotropic transition by *Stenotrophomonas bentonitica*

**DOI:** 10.1007/s11356-024-34256-z

**Published:** 2024-07-12

**Authors:** Miguel Angel Ruiz-Fresneda, Guillermo Lazúen-López, Eduardo Pérez-Muelas, Jesús Peña-Martín, Raúl Eduardo Linares-Jiménez, Antonio Martín Newman-Portela, Mohamed Larbi Merroun

**Affiliations:** 1 Department of Microbiology, University of Granada, Campus Fuentenueva, 18071 Granada, Spain; 2 Department of Human Anatomy and Embryology, Faculty of Medicine, University of Granada, 18016 Granada, Spain; 3 Centre for Biomedical Research (CIBM), Biopathology and Regenerative Medicine Institute (IBIMER), University of Granada, 18100 Granada, Spain; 4 Present Address: Institute of Resource Ecology, Helmholtz-Zentrum Dresden-Rossendorf, Dresden, Germany

**Keywords:** Selenate, Bioremediation, Multi-modal, Reduction, Thioredoxin, Flagellin

## Abstract

Microorganisms can play a key role in selenium (Se) bioremediation and the fabrication of Se-based nanomaterials by reducing toxic forms (Se(VI) and Se(IV)) into Se(0). In recent years, omics have become a useful tool in understanding the metabolic pathways involved in the reduction process. This paper aims to elucidate the specific molecular mechanisms involved in Se(VI) reduction by the bacterium *Stenotrophomonas bentonitica*. Both cytoplasmic and membrane fractions were able to reduce Se(VI) to Se(0) nanoparticles (NPs) with different morphologies (nanospheres and nanorods) and allotropes (amorphous, monoclinic, and trigonal). Proteomic analyses indicated an adaptive response against Se(VI) through the alteration of several metabolic pathways including those related to energy acquisition, synthesis of proteins and nucleic acids, and transport systems. Whilst the thioredoxin system and the Painter reactions were identified to play a crucial role in Se reduction, flagellin may also be involved in the allotropic transformation of Se. These findings suggest a multi-modal reduction mechanism is involved, providing new insights for developing novel strategies in bioremediation and nanoparticle synthesis for the recovery of critical materials within the concept of circular economy.

**Graphical Abstract:**

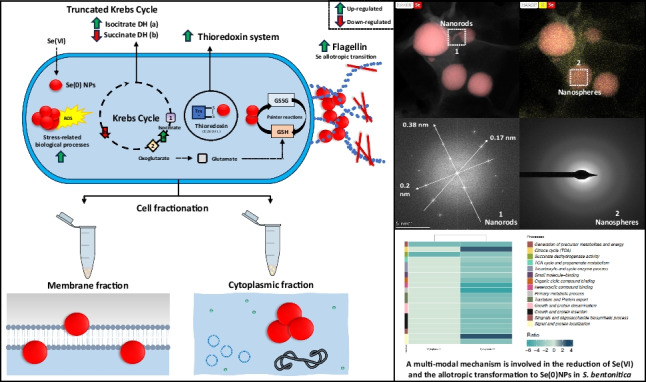

**Supplementary Information:**

The online version contains supplementary material available at 10.1007/s11356-024-34256-z.

## Introduction

Green technology innovation has been gaining attention in recent decades as an effective way to minimize adverse environmental impacts and achieve sustainable development. The fabrication of metallic nanoparticles (NPs), traditionally based on the use of hazardous materials, is a clear example. Novel biological procedures are being studied in depth with the aim of overcoming the limitations of physical–chemical methodologies. Bacteria, fungi, archaea, and plants have been widely reported for their capacity to produce gold (Au), silver (Ag), selenium (Se), tellurium (Te), lead (Pb), and zinc (Zn) NPs, amongst others, which are of special interest in many industrial applications (Ruiz-Fresneda et al. 2023a; Singh et al. 2018). Many bacterial species can enzymatically reduce toxic oxyanions of Se (selenite/Se(IV) and selenate/Se(VI)) producing elemental Se (Se(0)) in the form of NPs (Kora 2018; Tugarova et al. 2020; Ojeda et al. 2020). Thus, bacteria are suitable candidates for bioremediation purposes and for the environmentally-friendly synthesis of tailored SeNPs of industrial and medical interest, providing a potentially important role within the circular economy concept.

These oxidized and water-soluble forms of Se can be bio-reduced through several pathways. In assimilatory reduction Se is incorporated into amino acids (selenocysteine and selenomethionine) in the form of selenides, forming selenoproteins of essential importance in the correct functioning of the bacterial metabolism. For instance, selenocysteine is known to be crucial in the catalytic activity of enzymes such as thioredoxin reductase or glutathione peroxidase (Hawkes and Alkan 2010). The assimilatory reduction is expected to play a minor role in the bioremediation of seleniferous habitats due to the small Se fluxes involved in comparison to dissimilatory reactions (Eswayah et al. 2016). Dissimilatory (respiratory) reduction can support growth via anaerobic reduction of microorganisms obtaining metabolic energy using Se oxyanions as electron acceptors. However, Se may also be toxic for the cells due to its insertion into sulphur-containing proteins as Se and S share similar chemical properties. Both aerobic and anaerobic bacteria have adapted several detoxification processes to overcome this toxicity through its reduction into insoluble Se(0). Different mechanisms have been proposed for microbial detoxification via the thioredoxin system, Painter-type reactions involving thiol groups, siderophore-mediated reduction, and volatilization/methylation (Shimizu et al. 2021; D. Wang et al. 2019). Some of these potential mechanisms can occur inside the cell (Painter and thioredoxin reactions), whilst others may occur extracellularly (siderophore-mediated reduction). The Se reduction products derived from dissimilatory reduction and detoxification are generally Se(0) in form of NPs and selenides such as hydrogen selenide (H_2_Se), dimethyl diselenide (DMDSe), or dimethyl selenenyl sulphide (DMSeS) (Ruiz-Fresneda et al. 2020a; Eswayah et al. 2016). A significant distinction between selenium-respiring bacteria and Se detoxifying microbes lies in the externalization of the produced Se(0) NPs. The mechanisms for releasing these SeNPs in selenium-respiring bacteria have been observed to occur through vesicle mediated transport, as opposed to detoxification mechanisms which result in the formation and externalization of large-sized SeNPs through cell lysis mechanisms.

Similar to all the biochemical processes involved in bacterial metabolism, all these reduction processes are generally mediated by proteins and enzymes from different cellular compartments. Although many bacteria have been described for their ability to reduce Se(IV) by all these mechanisms, not so many have been found to reduce Se(VI). Se(VI)-reducing bacteria carry out this process in two different steps (Kushwaha et al. 2022). Firstly, Se(VI) must be transported into the cells and reduced to Se(IV) mainly through selenate reductases. Then, the formed Se(IV) is reduced to Se(0) by non-specific selenite reductases. A few procedures for transporting Se into the intracellular space have been described in the literature. Mainly sulphate transporter proteins are involved in selenate uptake in bacteria due to the physico-chemical and structural similarities of Se oxyanions and sulphur (Reich and Hondal 2016). For instance, an ATPase sub-unit (encoded by *cysA* gen) of the SulT sulphate permease was demonstrated to be crucial in Se(VI) reduction conducted by *Comamonas testosteroni* S44 (Tan et al. 2018). Once inside the cell, Se(VI) can be reduced to Se(IV) and finally Se(0) through selenate and selenite respiratory and non-respiratory reductases. *Thauera selenatis* was the first bacterium to be recognized that could use Se(VI) as a final electron acceptor in anaerobic respiration (Macy et al. 1993). This bacterium uses SerABC selenate reductase, a heterotrimeric enzyme (sub-units SerA, SerB, and SerC) specific for Se(VI) located in the periplasm. Other selenate reductases have also been found in the cytoplasmic membrane of *Enterobacter cloacae* SLD1a-1 and *Sulfospirillum barnesii*, and in the cytoplasm of *E. coli* K-12 (Bébien et al. 2002; Oremland et al. 1999; Ridley et al. 2006). However, more is known about the subsequent Se(IV) reduction conducted by proteins such as thioredoxin reductase, glutathione reductase, glutathione peroxidase, or fumarate reductase from different bacterial isolates (Debieux et al. 2011; Hunter and Kuykendall 2007; Lampis et al. 2017; Song et al. 2017).

The aim of this study is to better understand the mechanisms involved in the reduction of Se(VI) by the bacterium *S. bentonitica*. Although there are several mechanisms described for Se(IV) bio-reduction, there is still a significant knowledge gap in the mechanistic understanding of the processes involved in Se(VI) reduction that needs to be addressed. This strain was previously demonstrated to reduce Se(VI) into crystalline Se(0) nanorods after 24 h, but only at concentrations higher than 100 mM (Ruiz-Fresneda et al. 2023b). Previous transcriptomic analysis using whole cells of this strain revealed the regulation of oxidoreductases including nitrate reductases and selenium transporters, which can play an important role during the reduction process (Pinel-Cabello et al. 2023). To go further, in this study, the protein content of the different sub-cellular fractions was brought into contact with Se(VI) and individually analysed using proteomics and complementary spectroscopic and microscopic methodologies. We have been able to provide evidence for the existence of different metabolic pathways involved in Se(VI) reduction. In addition, we have identified several key enzymes which could play an important role in this process. For this reason, our results could be very useful in the development of new biotechnological bioremediation and tailored nanoparticle synthesis strategies.

## Materials and methods

### Bacterial strain and culture conditions

*Stenotrophomonas bentonitica*, a rod-shaped, Gram negative, and aerobic bacterium used in this study, was isolated from bentonite clay formations from southern Spain (Sánchez-Castro et al. 2017). This bacterium has previously been reported for its tolerance to a wide spectrum of heavy metals and metalloids such as selenium (Se), uranium (U), curium (Cm), and europium (Eu) (López-Fernández et al. 2014; Ruiz Fresneda et al. 2018; Ruiz-Fresneda et al. 2019, 2020a, 2020b; Sánchez-Castro et al. 2017). Additionally, the whole genome of *S. bentonitica* has been sequenced recently and it is available online (GenBank accession number MKCZ00000000) (Sánchez-Castro et al. 2017). The cells were grown in Lysogeny Broth (LB) medium containing 10 g/L tryptone, 5 g/L yeast extract, and 10 g/L sodium chloride with pH 7.0 ± 0.2 at 28 °C.

### Selenium solution preparation

Selenate stock solutions were prepared at 1 M concentration by dissolving sodium selenate (Na_2_SeO_4_) (Merck Sigma-Aldrich) in distilled water. After that, the stocks were sterilized by filtration using 0.22 µm syringe filters. These solutions were diluted to the desired concentration prior to use in the experiments.

### Sub-cellular fractionation and protein extraction

The cellular fraction separation and protein extraction were carried out to locate the enzymatic Se(VI) reduction activity of *S. bentonitica* cells. For this purpose, the procedures of Sandrini et al. (2014) were followed with some modifications. Briefly, a total of 1200 ml of *S. bentonitica* culture were centrifuged at 7000 g and 4 °C for10 min. The supernatants were stored as secreted proteins. The resultant pellets were washed twice with saline solution (0.9%) and with 10 mM Tris–HCl (pH 7.5) and finally resuspended in 10 mM Tris-base (pH 7.5). The suspensions were then frozen overnight at − 80 °C to weaken the cell membranes and facilitate sonication. The cells were then lysed by sonication using a VCX-130 PB (Sonics & Materials) ultrasonic processor (4 °C). After centrifugation of the lysed cells under the same conditions mentioned previously, the supernatants containing the total protein extract (cytoplasmic and membrane proteins) were separated by ultracentrifugation at 108,000 g for 10 min at 4 °C using a Beckman L8-M ultracentrifuge. After this step, the supernatants contained the total cytoplasmic proteins, whilst the pellet contained the total membrane protein. Finally, the pellets were incubated in 10 mM Tris–HCl (pH 7.5) with 2% Triton X-100 for30 min at room temperature and ultracentrifuged again at 108.000 g for 10 min at 4 °C. The subsequent pellets containing the outer membrane protein fraction and supernatants containing inner membrane proteins were stored at − 80 °C.

Total protein quantification of the sub-cellular fractions was carried out by the Invitrogen™ Qubit™ 3 fluorometer using the Qubit Protein Assay Kit (Invitrogen™).

### Selenium-reducing activity assays

After cellular fractionation and protein extraction, the Se(VI)-reducing capability of each fraction (cytoplasm, total membrane, inner and outer membrane) was evaluated. The evaluation involved incubating them with Se(VI) (200 mM) in the same buffer used for the extraction (Tris–HCl 10 mM—pH 7.5), in both the presence and absence of NADH (10 mM) as an electron donor. This elevated Se(VI) concentration was selected based on previous findings (Ruiz-Fresneda et al. 2023b; Pinel-Cabello et al. 2023). Basically, a discernible shift to a reddish hue only occurred at concentrations surpassing 100 mM of Se(VI) (Ruiz-Fresneda et al. 2023b). At lower concentrations, Se(VI) does not exhibit toxicity towards *S. bentonitica*, as it does not inhibit bacterial growth (Ruiz-Fresneda et al. 2023b)*.* Negative controls containing NADH and protein buffer in the presence of Se(VI) were prepared to confirm the reduction as a result of protein activity. Pure cell cultures of *S. bentonitica* in the presence and absence of Se(VI) were prepared as positive controls. A summary of the different treatments is shown in Supplementary Table 1. Finally, the Se reduction activity assays were conducted under agitation at room temperature on 1 ml Eppendorf® tubes to a final volume of 0.5 ml. The protein concentration used for the experiments was set up at 0.5 mg/ml. All the assays were prepared in duplicate.

### High-angle annular dark-field scanning transmission electron microscopy (HAADF-STEM)

We used electron microscopy for the structural elemental characterization of the Se reduction products and proteins presented in our samples. The collected sub-cellular fractions of *S. bentonitica* were supplemented with Se(VI) (200 mM) as mentioned in “Selenium-reducing activity assays” for 216 h and 1 month, and prepared for electron microscopy using the rapid, easy, and conventional negative staining (NS) procedure with uranyl acetate following the procedures of Ruiz-Fresneda et al. 2020a. NS is commonly used for protein characterization and interactions. The samples were examined under a HAADF-STEM microscope THERMO FISHER TALOS F200X equipped with energy dispersive X-ray (EDX), selected area electron diffraction (SAED), and fast Fourier transform (FFT) for compositional and structural characterization. STEM specimen holders were cleaned by plasma prior to STEM analysis to minimize contamination.

### Attenuated total reflection–Fourier transform infrared (ATR-FTIR) spectroscopy

In order to determine the possible functional groups bound to the SeNPs, and thus potentially involved in the reduction of Se(VI) and/or stabilization of the reduction products, the infrared spectra of the cytoplasm and total membrane treatments were analysed. For this purpose, the samples were harvested after 216 h incubation and subjected to centrifugation (11,000 × g, 10 min). Subsequently, the precipitates were washed 3 times with distilled water and dried at 28 °C.

ATR-FTIR measurements were performed on a JASCO 6200 spectrometer, equipped with a JASCO PRO ONE ATR accessory, consisting of a diamond crystal at a fixed angle of 45°. Fifty scans at 4 cm^−1^ spectral resolution and wave-number range from 4000 to 400 cm^−1^ were collected for each sample. All the measurements were in triplicated. Spectra obtained were baseline corrected, smoothed, and normalized to 1.5 absorbance (arbitrary units) using the 1650 cm^−1^ amide I absorption band from the control samples (sub-cellular fractions without Se and NADH). Finally, the acquisition and processing of the data were performed using the Spectra Manager version 2.13.00 software.

### Nano-LC–MS/MS

To separate the proteins by molecular weight (kDa) within a given collected fraction, the samples were loaded in SDS–polyacrylamide gel electrophoresis (SDS-PAGE). All the cytoplasmic and total membrane based treatments were analysed to determine the differences in their protein profile in the presence of Se(VI) and NADH, and hence the potential proteins involved in the Se(VI) reduction process. Firstly, the samples (0.5 mg/ml final concentration) were mixed with 2 × loading buffer containing 0.1 M Tris–HCl (pH 6.8), 4 mM EDTA pH 8, 2% SDS, 25% glycerol, 0.01% bromophenol blue, and 10% β-mercaptoethanol. Subsequently, the proteins were denatured at 90 °C for 10 min and loaded into a 12% (w/t) 1.0 mm polyacrylamide SDS-PAGE. The Blue Easy Prestained Protein Marker (NIPPON Genetics) was loaded in the same gel as the protein marker. Electrophoresis was performed at room temperature for approximately 1 h using a constant voltage (200 V) in a 1 × running buffer solution containing SDS (0.5%), glycine (7.2%), and Tris-Base (1.5%). The gels were stained by incubating them for 30–60 min on agitation (50 rpm) into a staining buffer solution containing 0.025% Coomasie blue (Brilliant Blue G—Sigma Aldrich), 45% ethanol, 5% glacial acetic acid, and the rest of the distilled water. Finally, before visualization, the gels were incubated in a destaining buffer containing 10% 2-propanol, 10% glacial acetic acid, and the rest of distilled water for 1 h at 50 rpm with agitation.

Protein spots observed in the SDS-PAGE gel were carefully excised and further processed for their identification by Nano-LC–MS/MS. Firstly, the excised bands were subjected to dithiothreitol (DTT), iodoacetamide (IAM), and trypsin (enzyme:substrate ratio 1:40) digestion for 18 h at 30 °C. The resulting pellets containing the peptides were extracted by using 0,2% TFA (trifluoroacetic acid) and 30% acetonitrile for 1 h. Subsequently, the eluted samples were dried using a SpeedVac system and stored at 20 °C. Finally, the samples were analysed by Nano-LC (EASY nanoLC-Proxeon) coupled to an ion trap mass spectrometer (Amazon Speed ETD-Bruker). Protein identification was carried out by searching in the software ProteinScape (Bruker) using the Mascot Programme (Matrix Science).

### Differential enrichment analysis of the proteomics data

Integrated analysis of mass spectrometry proteomics data was carried out using the DEP package (Zhang et al. 2018) from the R statistical software (R Core Team 2022). Missing values from raw data were filtered and then attributed using different strategies according to the class of the missing data: missing at random (MAR) or not missing at random (MNAR). Briefly, only proteins identified in a minimum of two out of the three replicates of at least one condition were accepted. The remaining missing values were treated as MNAR when they were present in the three replicates of one condition but not present in the other, or treated as MAR when they were present in one the replicates of any condition. The MNAR values were attributed using random draws from a Gaussian distribution centred around a minimal value, and the MAR values were attributed using the *K*-nearest neighbour algorithm. The resulting data was then normalized by variance stabilising transformation and the contrasts between the conditions tested and filtered by FDR < 0.05 and logFC > log_2_(1.5).

### Biological process enrichment analysis (GO enrichment analysis) through STRING

The protein sequencing data from SDS-PAGE gel bands, previously subjected to cleavage and processing, were incorporated into Cytoscape, where an enrichment table was generated using the StringApp plugin and the redundant terms were filtered across six categories: GO Biological Process, GO Molecular Function, GO Cellular Component, KEGG Pathways, PFAM, and InterPro protein domains (Doncheva et al. 2019). The resulting dataset from this comprehensive analysis was then visualized using the EnrichmentMap plugin (Merico et al. 2010), with a Jaccard distance cutoff of 0.8. Additionally, the STRING database (Search Tool for the Retrieval of Interacting Genes) (Szklarczyk et al. 2019) was used for conducting a GO (Gene Ontology) term enrichment analysis. This analysis aimed to identify enriched GO terms associated with proteins that were either exclusive to Se-supplemented treatments or common proteins that showed an emPAI ratio > 1.5, and thus potentially belonging to the mechanisms activated by the cell after the Se exposure. For this purpose, their respective NCBI accession IDs were introduced into the STRING database and the search parameters were configured to encompass the “Full STRING network,” with a minimum confidence > 0.400 and FDR < 0.05.

## Results and discussion

### Sub-cellular localization of selenate reduction

**Fig. 1:**
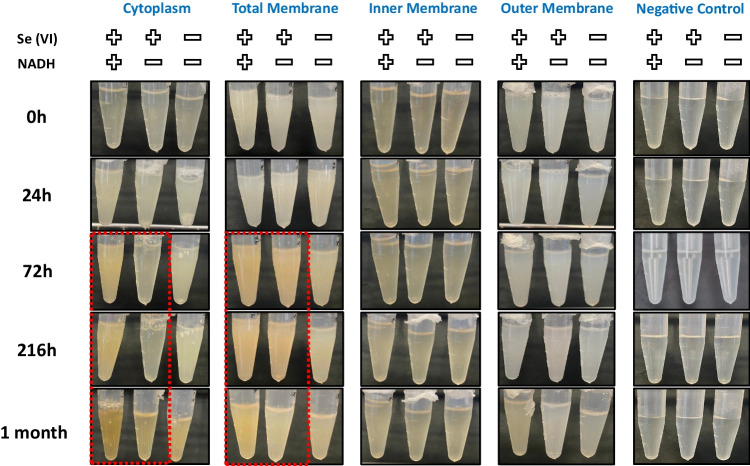
Se(VI) reduction assays of the different sub-cellular fractions of *S. bentonitica* (cytoplasm, total membrane, inner membrane, and outer membrane) in the presence and absence of Se(VI) and NADH. Protein buffers in the presence and absence of both Se(VI) and NADH were used as negative controls

To elucidate how Se(VI) is reduced by the cells of *S. bentonitica*, as previously indicated by the studies of Ruiz-Fresneda et al. (2023b), we extracted their sub-cellular fractions and contacted with Se(VI). Bioreduction of Se(VI) to Se(0) is typically manifested by the appearance of a light reddish colour in cell cultures. In our study, we observed that Se(VI) reduction activity by *S. bentonitica* occurs in both cytoplasmic and total membrane fractions as indicated by red precipitates (after 72 h), as can be seen in both the absence and presence of NADH (Fig. 1). However, the intensity of the red colouration seems to be slightly higher in those NADH-containing fractions. This suggests that NADH could enhance Se reduction serving as electron donor for reductase enzymes, which may have an important role in the Se biotransformation. Similar results were obtained by other bacterial strains such as *Proteus mirabilis* QZB-2 and *Enterobacter cloacae* Z0206 in cell membrane fractions supplemented with NADH/NADPH (Huang et al. 2022; Song et al. 2017). In the case of the bacterium *Stenotrophomonas maltophilia* SeITE02, only the cytoplasmic fractions triggered Se(IV) reduction exclusively in the presence of NADH, suggesting the involvement of enzymatic mechanisms during the process (Lampis et al. 2017). However, the reduction in the absence of NADH in our samples indicates additional molecular mechanisms may be involved.

The inner and outer membrane fraction showed no reduction of Se(VI) as it remained colourless (Fig. 1). It seems the cell membranes (inner and outer) need to remain integrated and intact in order to carry out the Se(VI) reduction as indicated by the clear shift to red in the total membrane fractions in comparison to the inner and outer membrane samples. The appearance of red precipitations in the total membrane in comparison with the inner and outer membranes indicates that periplasmic proteins, which are mostly lost during the separation of the internal and external membrane, may have an important role in the reduction process. The non-formation of Se(0) red precipitates in the negative controls [protein buffer ± Se(VI) ± NADH] revealed the reduction of Se(VI) is conducted by the cell fractions and not by NADH or the buffer components (Fig. 1). Many other authors have documented the Se(IV)-reducing activity of cellular sub-fractions from different bacterial strains including the aforementioned *E. cloacae* Z0206, *S. maltophilia* or *P. mirabilis*, and others such as *Bacillus cereus*, *Proteus penneri* LAB-1, and *Burkholderia fungorum*. (Khoei et al. 2017; Kora 2018; Wang et al. 2022). However, to the best of our knowledge, there are no reports on Se(VI) reduction by bacterial sub-cellular fractions.

### Microscopic characterization of the Se reduction products

The Se reduction products derived from the different sub-cellular fractions were structurally characterized by HAADF-STEM for the samples which showed a deep red colour (after 216 h and 1 month incubation period). No significant differences were observed between the two incubation times analysed. Moreover, no significant differences were appreciated in those samples supplemented with NADH.

#### Cytoplasmic fraction

**Fig. 2:**
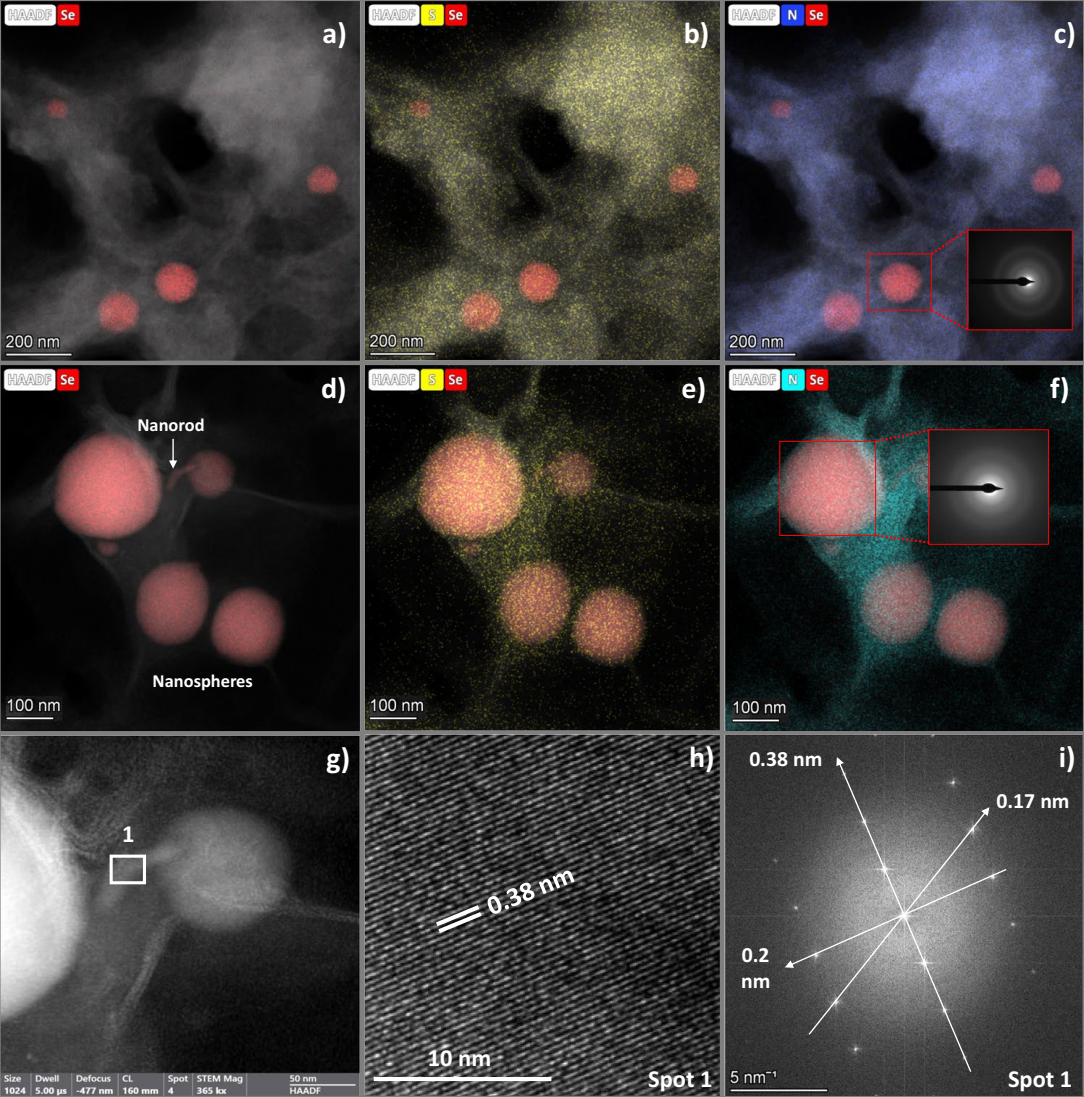
HAADF-STEM micrographs with their elemental distribution mapping of the cytoplasmic fraction of *S. bentonitica* showcasing Se nanospheres embedded within an organic matrix primarily composed of S and N (**a**–**c**). HAADF-STEM images and element-distribution mapping of the total membrane fraction of *S. bentonitica* revealing Se nanospheres and nanorods enclosed in an organic matrix containing S and N (**d**–**f**). SAED pattern from a single Se nanosphere in the cytoplasmic fraction (panel inset in **c**) and the total membrane fraction (panel inset in **f**). High-resolution image (**h**) and FFT pattern (**i**) derived from a single nanorod (spot 1 in **g**)

Microscopy images from the cytoplasmic fraction of *S. bentonitica* contacted with Se(VI) showed very few Se electron-dense nanospheres ranging from 50 to 200 nm (Fig. 2a-c). They were embedded in an organic matrix with signals of sulphur (S) and nitrogen (N) as indicated by EDX (energy-dispersive X-ray) elemental mapping (Fig. 2b–c). These results suggest the role of sulphur-containing proteins in the formation of SeNPs. Indeed, a great number of S-containing enzymes, such as thioredoxin reductase, glutathione reductase, and glutathione peroxidase, have previously been identified as being involved in the reduction of Se oxyanions and the formation of Se(0) (Hunter and Kuykendall 2007; Lampis et al. 2017). Interestingly, all the NPs presented an amorphous nature as shown by the SAED patterns (Fig. 2c).

#### Total membrane fraction

Microscopy images from the total membrane fraction of *S. bentonitica* supplemented with Se(VI) also showed the presence of many electron-dense nanospheres with a similar size (from 50 to 200 nm) associated to a thick organic matrix (Fig. 2d). Very few small (around 20 nm thick) tubular-shape nanoparticles (NPs) were also found as can be seen in Fig. 2g. EDX elemental mapping analysis confirmed the nanospheres, and the tubular NPs were made of Se with trace amounts of S and N (Fig. 2e–f). Interestingly, a higher number of SeNPs were found compared to the cytoplasmic fraction. This may indicate a more important role of membrane proteins of *S. bentonitica* in the reduction and SeNP synthesis process. Spherical NPs exhibited an amorphous nature as indicated by SAED patterns (Fig. 2f). However, high-resolution images in combination with FFT allowed us to detect different crystalline d-spacings in some NPs (Fig. 2h–i). Specifically, three different lattice spaces were estimated: 0.176 nm, 0.2 nm, and 0.38 nm. All the d-spacings obtained may correspond to different growth planes of both monoclinical (*m*-Se) and trigonal (*t*-Se) according to the American Mineralogist Crystal Structure Database. The lattice space of 0.38 nm can be attributed to crystal planes of t-Se (100), or gamma m-Se (022) and alpha m-Se (− 121). 0.2, and 0.176 nm can also be related to different *t*-Se and *m*-Se crystalline planes. So, in this sub-cellular fraction, both amorphous and crystalline Se were formed.

#### Inner and outer membrane fractions

The Se-reducing assays suggest the inner and outer membranes of *S. bentonitica* cells do not reduce Se(VI) as no red accumulations were appreciated in the Se-containing samples (Fig. 1). HAADF-STEM micrographs confirmed this as no SeNPs could be observed in either the presence or absence of NADH (Supplementary Fig. 1).

#### Sub-cellular fraction controls

None of the sub-cellular fractions controls in the absence of Se(VI) and NADH analysed by STEM showed any Se form or structure as can be observed in micrographs in Supplementary Fig. 2. This observation together with non-formation of red precipitates in all the negative controls (Fig. 1) confirmed the SeNP formation is conducted by each protein fraction.

#### The role of the different sub-cellular compartments in Se reduction and allotropic transformation

Several authors have investigated the Se(IV)-reducing ability of the various sub-cellular fractions of different bacterial strains; however, just a few studies have focused on Se(VI). Wang et al. (2022) reported that the total membrane fraction of *Proteus penneri* Lab1 showed Se(IV) reduction activity in the presence and absence of NADH. Huang et al. (2021) described that Se(IV) reducing-ability of *Providencia rettgeri* HF16-A was mediated by cytoplasmic proteins only when NADH or NADPH was present. They suggested that Se(IV) may be reduced to Se(0)NPs through several cytoplasmic reductases with NADH/NADPH serving as electron donor, and their subsequent release to the extracellular space. In our study, we have conducted the Se(VI) reduction in both total membrane and cytoplasmic fractions regardless of the presence or absence of NADH, which suggests a multi-modal reduction mechanism. The intracellular Se(VI) reduction may consist of a first Se(VI) uptake step followed by reduction and formation of Se(0) NPs phase. In addition, Se(VI) reduction activity we also observed in both the total membrane, indicating that Se(VI) can either pass through or attach to the membrane. This suggests that Se(VI) can be reduced to Se(0) NPs in the periplasm concurrently with intracellular reduction.

A Se allotropic transformation process from amorphous Se (*a*-Se) nanospheres to stable trigonal (*t*-Se) SeNPs crystals with different shapes (nanorod, hexagons, polygonal-shaped) has been previously proposed in *S. bentonitica* whole cells in the presence of Se(IV) (Ruiz-Fresneda et al. 2018). They suggested that *a*-Se nanospheres are formed intracellularly during the first 24 h and after that they are released outside and start to agglomerate until they are converted into Se crystals after 144 h. However, when Se(VI) was used as initial Se oxyanion, only the formation of big *t*-Se nanorods were observed in just 24 h of incubation (Ruiz-Fresneda et al. 2023b). A possible explanation for this is that the lower toxicity of Se(VI) accelerated the transformation process and only crystalline *t*-Se nanorods were observable (Ruiz-Fresneda et al. 2023b). The production of Se(0) crystals is interesting from an economic point of view, as they exhibit attractive properties compared to *a*-Se, including, for example, high photoconductivity and piezoelectricity (Ren et al. 2012). Interestingly, in the cytoplasmic and total membrane sub-cellular fraction images of *S. bentonitica* here presented, mostly *a*-Se nanospheres were observed after 216 h and 1 month of incubation. Only a few Se crystals could be detected in the total membrane samples. Although the previously mentioned sub-cellular fractions are capable of reducing Se(VI) to *a*-Se(0) NPs, the crystallization rate is significantly slower in comparison to the fully functional whole cells. It seems that the whole cells and hence, the complete physiological and cellular activity are necessary for an effective and complete morphological transformation and crystallization. To sum up, all these results suggest that Se reduction can occur by multiple mechanisms in different compartments; however, Se crystallization is a more complex process and the whole operating cell is needed for an effective and quick allotropic transformation.

### SeNP characterization by ATR-FTIR

**Fig. 3:**
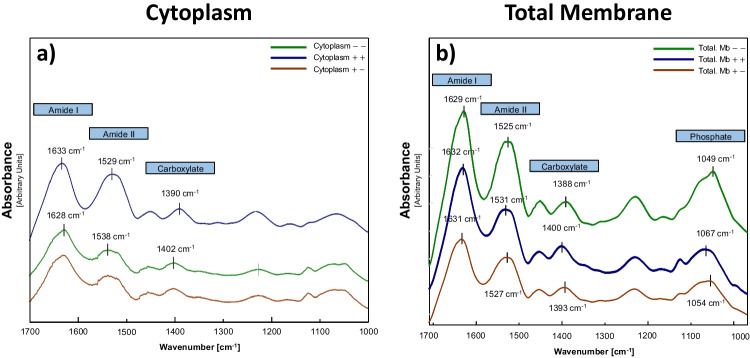
Comparison between the ATR-FTIR spectra of different subcellular fractions of the bacterium *S. bentonitica* in the presence and absence of both Se(VI) and NADH after 216 h. **a** Cytoplasmic and **b** total membrane protein extracts from *S. bentonitica* incubated with 200 mM Se(VI) and 10 mM NADH (in blue colour), without NADH (in orange colour) and in the absence of both Se(VI) and NADH (in green colour). Note that the infrared spectrum generated by the cytoplasm fraction without Se(VI) and NADH is identical to the treatment of the same fraction supplemented with Se(VI) but without NADH (in green colour)

We conducted ATR-FTIR analysis to better characterize the Se-protein extract interactions by identifying the functional groups involved. Whilst the spectral region spanning from 4000 to 1800 cm^−1^ did not exhibit significant changes in the absorption bands among the different treatments (data not shown), distinct signal shifts were observed in the 1700–1000-cm^−1^ region for the spectra of both the membrane and cytoplasm fractions in comparison to their respective controls (Fig. 3). Specifically, one minor shift was observed at around 1538 cm^−1^ for both cytoplasmic and total membrane fractions, caused by N–H bending (*δ*_N-H_) and the contributions arising from stretching C-N (*ν*_C-N_) groups, which are mainly associated with the amide II band (Barth 2007). Another notable shift was found at 1400 cm^−1^, consistent with the symmetric C-O stretching of the carboxylate groups (*ν*_sym COO_-) (Ojeda et al. 2008). The significant shifts observed in the absorption bands of the carboxylate groups, coupled with the minor shifts in the absorption bands of amide II, may indicate a strong binding of Se to the carboxyl groups of the proteins. This binding could lead to an alteration in the protein folding configuration, particularly considering that changes in the amide II bands have been linked to a loss of the secondary structure of the protein (Oberg et al. 2004).

Significant differences were observed between the cytoplasmic fraction patterns with added NADH (+ +) and those without NADH addition ($+-$ and $--$) (Fig. 3a). This fact, along with the higher production of SeNPs and organic matrix coating in the presence of NADH, supports the hypothesis that cytoplasmic reduction is highly dependent on NADH activity. Some modifications in bands attributed to the double bond stretching of > P═O of general phosphoryl groups and phosphodiester of nucleic acids at around 1070 cm^−1^ (Hufton et al. 2016) were only identified in the total membrane fraction (Fig. 3b). This shift could be indicative of a potential novel mechanism of Se binding to phosphate groups of phospholipids from the bacterial membrane. In conclusion, our results provide robust evidence supporting the presence of an interaction between Se with the bacterial membrane and cytoplasmic proteins. These observations strongly suggest the existence of a multifaceted network of interaction mechanisms, consistent with the patterns observed in our microscopy results.

### Identification of proteins involved in Se(VI) reduction by *S. bentonitica*

During the proteomic analysis of cytoplasmic and total membrane fractions, we observed that some proteins typically associated with the total membrane were detected in the cytoplasmic fraction, and vice versa. This may be attributed to the inefficiency of the sub-cellular fractionation process, allowing the detection of cytoplasmic and membrane proteins in both fractions. In fact, many current research efforts are being focused on designing effective methods for protein separation technologies (Liu et al. 2020). Moreover, all proteins, including membrane proteins, are synthesized in the cytoplasm before being directed to their respective locations. Therefore, they can be detected in the cytoplasmic fractions.

#### Overview of proteome analysis

One-dimension SDS-PAGE gel revealed differences in band intensity and protein profile for the different total membrane and cytoplasmic treatments (+ Se(VI) + NADH; + Se(VI) $-$ NADH; and $-$ Se(VI) $-$ NADH) (Supplementary Fig. 3). Subsequently, all the bands were subjected to LC–MS/MS for protein identification and comparison. The nano-LC–MS/MS examination of all the samples (replicates and controls included) generated a total of 2285 MS/MS spectra, of which 967 could be related to peptide sequences of the *S. bentonitica* BII-R7 proteome, complying with a p-value ≤ 0.05. This high assignment rate, representing 42.3%, indicates the high quality of the MS/MS spectra. The similarity of the data observed between the different replicates reflects the high reproducibility of the experimental procedure.

**Fig. 4:**
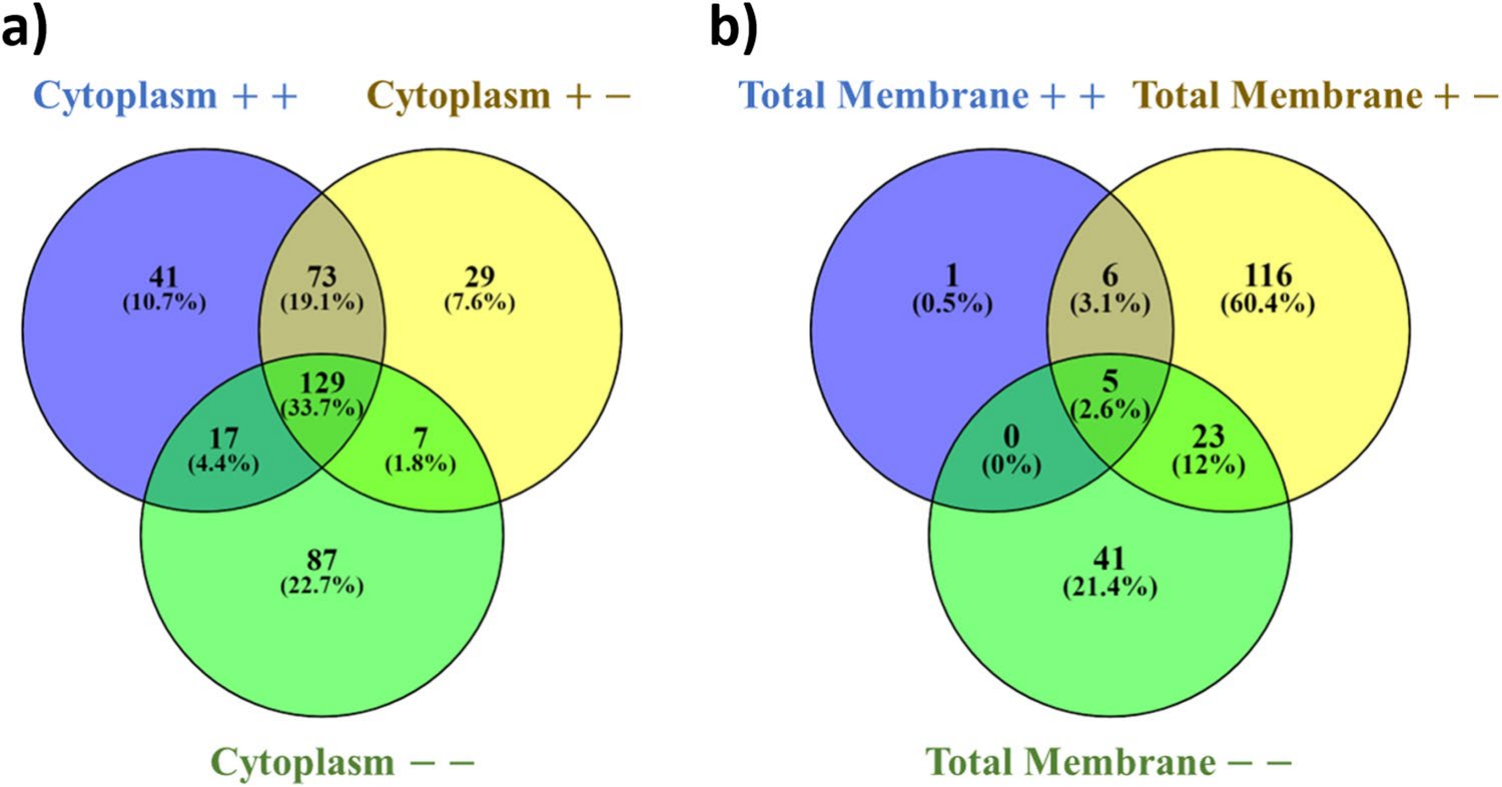
Venn diagram showing common proteins between different treatments: + Se(VI) + NADH in purple ($++$), $+$ Se(VI) $-$ NADH in yellow ($+-$), and the negative control $-$ Se(VI) $-$ NADH ($--$) in green from the cytoplasmic (**a**) and total membrane fraction (**b**)

In the cytoplasmic fraction, we observed that only 33.7% (129 out of 423) of the sequenced proteins were common to all the treatments (Fig. 4a) evidencing a significant difference in the protein profile depending upon the addition of Se(VI) or/and NADH. This fact can also be appreciated by comparing Se(VI)-treated samples (cytoplasm $++$ and $+-$) with Se-untreated controls (cytoplasm $--$), as very few common proteins were detected (4.4% and 1.8%, respectively) (Fig. 4a). This indicates that Se(VI) significantly alters the protein metabolic response. Similarly, comparing the Se(VI)-treated fractions in the presence (cytoplasm + +) and absence of NADH (cytoplasm $+-$), a total of 52.8% shared proteins (19.1 + 33.7% = 52.8%) were observed. This suggests that different proteins maybe involved in Se(VI) mitigation depending on the addition of NADH.

In the case of the total membrane fraction, even more remarkable differences were observed. As seen in Fig. 4b, only 2.6% (5 out of 192) of the sequenced proteins were common to all treatment groups, showing a substantial remodelling of the core proteome when the addition of Se(VI) and NADH took place. This observation shows that the presence of Se(VI) leads to a more pronounced restructuring of the proteome within the total membrane fraction in comparison to the cytoplasmic fraction. Such extensive remodelling strongly supports the possibility that Se(VI) reduction could predominantly take place via the total membrane fraction. This fact agrees with the higher production of SeNPs observed in the total membrane fraction as was shown by TEM analysis. Thus, these results indicate, as in “Sub-cellular localization of selenate reduction” and “Microscopic characterization of the Se reduction products,” that the reduction process involves a multi-modal mechanism occurring in both the total membrane fraction and the cytoplasm. The versatility of *S. bentonitica* in terms of Se(VI) tolerance pathways grants it greater potential compared to other microorganisms for exploring new applications in bioremediation and nanotechnology.

#### Proteome response in the cytoplasmic fraction of *S. bentonitica*

**Fig. 5:**
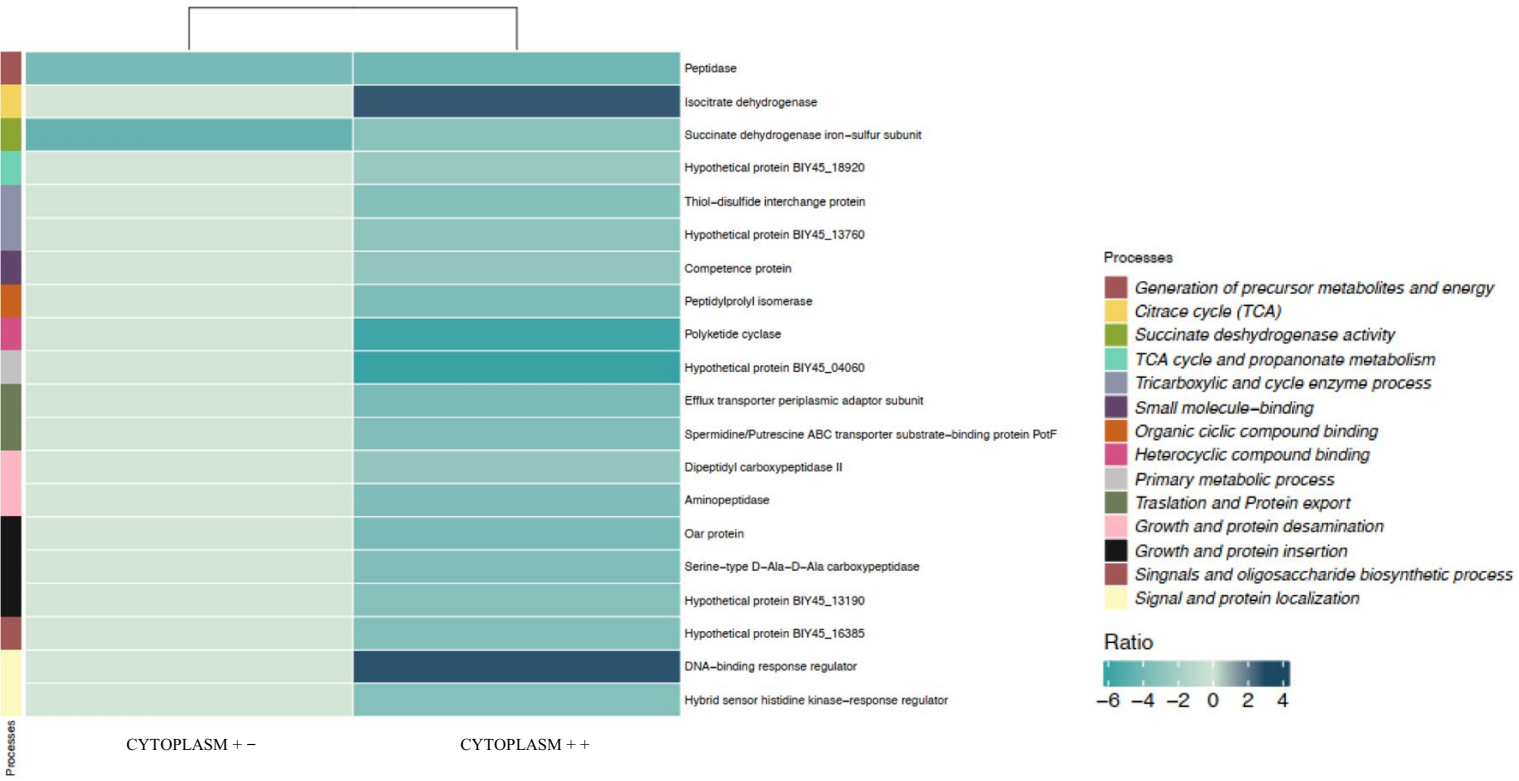
Heat map visualization of the relative emPAI Ratios of each significantly altered protein (cut off: 0.5%) between cytoplasm $++$ and cytoplasm $+-$ treatments. Various colour gradients illustrate the spectrum of ratio values, encompassing negative, zero, and positive values according to the scale displayed. On the right side, the different metabolic pathways associated with each protein are displayed. Note that the proteins not differentially expressed between treatments are assigned a value of 0 in the graph

Amongst the 17 common proteins (4.4%) analysed between the Se(VI) treated (cytoplasm $++$) and untreated cytoplasm (cytoplasm $--$) (Fig. 4a), only a small number exhibited a significantly different emPAI ratio. Specifically, there was a significant downregulation of two of them: succinate dehydrogenase (Ratio: $-$ 4.55) and peptidase (Ratio: $-$ 3.92) (Supplementary Table 2; Fig. 5). Both downregulated proteins are primarily associated with metabolic pathways related to energy acquisition, including the oxidative phosphorylation and the Krebs cycle (TCA-tricarboxylic acid cycle). Effectively, these processes are the most significantly altered according to the constructed metabolic pathway enrichment network (Supplementary Fig. 4A). Previous studies conducted by Avendaño et al. (2023) indicated a transcriptional downregulation of enzymes associated with TCA cycle when the strain *Pseudomonas putida* KT440 was exposed to 1 mM of Se(IV). They demonstrated that Se(IV) reduction is dependent on a combination of various metabolic pathways (sulphur metabolism, oxidative response, and oxoglutarate/glutamate) converging in the formation of glutathione, which can trigger the reduction mechanism. The negative cytoplasm $+-/--$ emPAI ratios found here for succinate dehydrogenase activity showed Se(VI) could interfere with the correct metabolic functioning associated with energy acquisition, resulting in a detrimental impact on the viability of *S. bentonitica* cells. However, the interruption of such metabolic routes could enhance the efficiency of Se(VI) reduction (Avendaño et al. 2023).

Cytoplasmic sub-cellular fraction amended with NADH and Se(VI) (cytoplasm $++$) showed higher protein profile alterations compared to the treatment without NADH (Fig. 5). This fact aligns very well with the results obtained from FTIR analysis in “SeNP characterization by ATR-FTIR,” which suggested the reduction in the cytoplasm is highly dependent on NADH metabolism. Indeed, significant differences in the cytoplasm $++/--$ emPAI ratios of several enzymes were observed, with some being upregulated and others downregulated. Specifically, among the 146 common proteins (129 + 17) present in both treatments (Fig. 4), 22 exhibited significant differences in their emPAI ratios. Some of the enzymes with more pronounced differential expression include succinate dehydrogenase (ratio: $-$ 3.21) and isocitrate dehydrogenase (ratio: 2.64) from the Krebs cycle, several peptidases involved in amino-acid synthesis and degradation such as serine-type D-Ala-D-Ala carboxypeptidase (ratio: $-$ 3.53), and various enzymes related to transport processes, including ABC transporter substrate-binding protein PotF (ratio: $-$ 3.54) and efflux transporter periplasmic adaptor subunit (ratio: $-$ 3.78) (Supplementary Table 2 and Fig. 5). Regarding the significantly upregulated proteins, thioredoxin was also identified as playing an important role with a positive emPAI ratio of 3.17. This protein has a function as an antioxidant by reducing other proteins, exchanging a thiol-disulphide group in cysteine (Yasir et al. 2020).

The upregulation of isocitrate dehydrogenase (emPAI ratio: 2.64) together with the downregulation of succinate dehydrogenase activity (emPAI ratio: $-$ 3.21) maybe indirectly involved in the Se(VI) reduction process by promoting the accumulation of oxoglutarate. The metabolic pathway enrichment network supported this suggestion as other processes controlling oxoglutarate formation and accumulation, such as the oxoglutarate dehydrogenase complex, oxocarboxylic acid, and glutamate metabolism, are significantly altered (Supplementary Fig. 4B). Oxoglutarate is the precursor for the synthesis of glutamate and glutathione, which are key metabolites for oxidative stress mitigation. Glutathione (glutamate-cysteine-glycine) is also involved in Se reduction through what are known as Painter-type reactions. These reactions involve the participation of thiol-containing molecules that can trigger the reduction process by forming precursor molecules (Nancharaiah and Lens 2015). The role of thiol-containing enzymes such as glutathione was initially suggested by EDX analysis obtained with HAADF-STEM and agrees herein through proteomics. Notably, Avendaño et al. (2023) and Zhang et al. (2016) described similar results, as a mutation in the D-2-hydroxyglutarate dehydrogenase (D2HGDH) gene, which encodes the enzyme involved in replenishing the pool of oxoglutarate, showed a deficient phenotype in the Se(IV) reduction process of *P. putida* KT2440.

Certain periplasmic transporters including the protein PotF (ABC transporter system) and efflux transporter periplasmic adaptor sub-unit, exhibited significant downregulation. These modifications on ABC transport and other efflux systems, whose primary function is cellular detoxification through the expulsion of toxic compounds (Igarasi et al. 2001), evidenced the cellular stress induced by Se(VI). The reduced activity of these transporters may enhance the intracellular Se(VI) reduction processes by decreasing its release into the extracellular space. This fact is consistent with the upregulation of certain oxidoreductases, which could be involved in the reduction process. Numerous enzymes have been described in the literature for their potential role in Se(VI) reduction. Although the first step of reduction from Se(VI) to Se(IV) is not well understood, more is known about the second step from Se(IV) to Se(0). Several permeases have been reported for Se(VI) uptake before being reduced through Se(VI) and Se(IV) oxidoreductases including glutathione reductase, thioredoxin reductase, and fumarate dehydrogenase (Debieux et al. 2011; Hunter Kuykendall 2007; Lampis et al. 2017; Song et al. 2017). For instance, Fischer et al. (2020) identified thioredoxin-disulphide and glutathione reductases to be involved in the biotransformation of Se(IV) to Se(0)NPs synthesized by *B. safensis* JG-NB5T. In our study, the upregulation of thioredoxin (ratio: 3.17) indicates its potential role in the Se(VI) reduction process. The thioredoxin system has been reported to participate in several redox reactions including Se(IV) reduction. In this process, reduced thioredoxin reacts with selenodiglutathione (GS-Se-SG) to form reduced glutathione (GSH), selenopersulphide (GS-Se^−^), and oxidized thioredoxin (Nancharaiah and Lens 2015). GS-Se^−^ is an unstable compound and undergoes a hydrolysis reaction to form GSH and Se(0). With the help of an electron donor [NAD(P)H], oxidized thioredoxin can be reduced by thioredoxin reductase thus starting the cycle again. However, thioredoxin reductase has also been reported to directly participate in Se reduction (Kumar et al. 1992; Shimizu et al. 2021). The existence of the thioredoxin system would lead not only to the reduction of Se(IV) but also to the formation of reduced glutathione (GSH) (Nancharaiah and Lens 2015). This would represent a new way of producing glutathione in addition to the one previously mentioned related to the truncation in the Krebs cycle.

#### Proteome response in the total membrane fraction of *S. bentonitica*

**Fig. 6:**
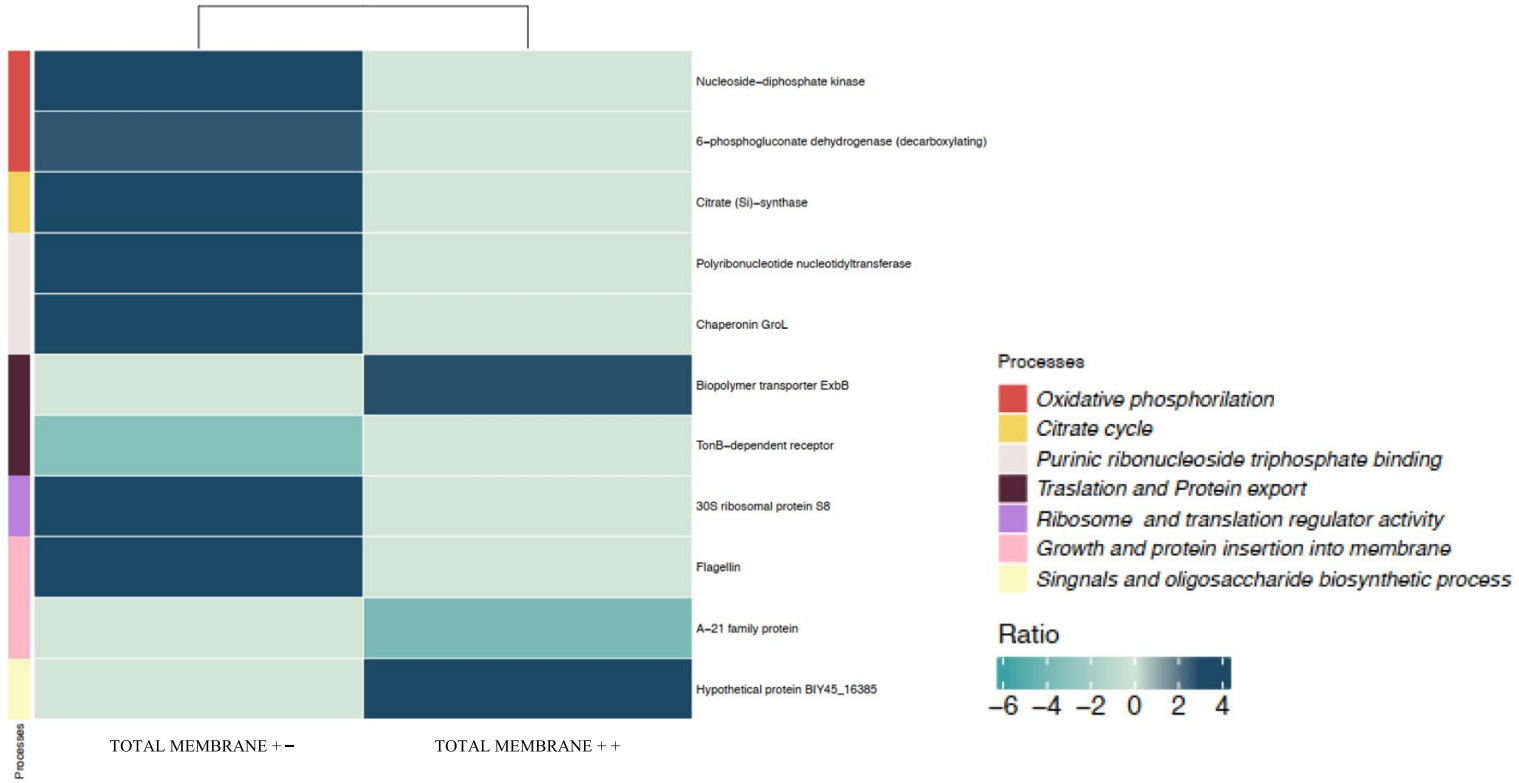
Heat map visualization of the relative emPAI ratios of each significantly altered protein (cut off: 0.5%) between total membrane $++$ and total membrane $+-$ treatments. Various colour gradients illustrate the spectrum of ratio values, encompassing negative, zero, and positive values according to the scale displayed. On the right side, the different metabolic pathways associated with each protein are displayed. Note that the proteins not differentially expressed between treatments are assigned a value of 0 in the graph

Total membrane treatment with Se(VI) and without NADH (total membrane $+-$) showed upregulation of proteins related to energy acquisition, such as malate dehydrogenase and citrate synthase, which exhibited an emPAI ratio of 4.01 and 3, respectively (Fig. 6; Supplementary Table 3). This fact differs from the ratios found in the cytoplasmic $+-$ protein extract, where the only significantly altered enzyme involved in the TCA cycle was downregulated (e.g. succinate dehydrogenase emPAI ratio: $-$ 4.55). The above mentioned enzymes play a crucial role during the TCA cycle by the synthesis of important metabolites (e.g. NADH) for energy production during oxidative phosphorylation, the final stage of cellular respiration. According to the results, it may be that the energy acquisition primarily takes place through a membrane mechanism such as oxidative phosphorylation and that the cytoplasmic metabolic processes are more focused on protecting the cells against Se(VI) through enzymatic reduction.

A notable downregulation of the protein transporter TonB (emPAI ratio: $-$ 3.27) was detected. TonB-dependent transporters have previously been reported as playing an important role under stress situations through the uptake of iron (siderophores), which is essential in many biological functions. Its role also involves the transport of other molecules including antibiotics and metals from the extracellular space to the periplasm and eventually into the cytoplasm (Noinaj et al. 2010; Pinel-Cabello et al. 2021). Additionally, an increase in the expression of enzymes involved in ribonucleoside phosphate metabolism, such as nucleoside diphosphate kinase (emPAI ratio: 3.53) and 6-phosphogluconate dehydrogenase (emPAI ratio: 2.71), has been detected (Fig. 6; Supplementary Table 3). These proteins are involved in the binding of nucleotides and small molecules, the synthesis of ribonucleotide monophosphates, and the mechanisms related to DNA synthesis and regulation (Supplementary Fig. 5A). Other fundamental processes for cellular function, such as the degradation and synthesis of RNA, regulation of translational activity, and protein export, also appear to be altered (Supplementary Fig. 5A). Therefore, it was again also possible to observe that Se(VI) significantly interferes in the proper functioning of the cell machinery.

The high emPAI ratio for flagellin is one of the most interesting findings reported here (emPAI ratio: 3.3) (Fig. 6; Supplementary Table 3). Flagellin proteins are primarily involved in motility functions, serving as structural components of flagella, and also in biofilm formation (Belas 2014). In the presence of stress agents, these functions could be helpful for defence against them or for the search for more favourable environments. Therefore, their upregulation can be interpreted as an enhancement of the defensive mechanisms. Although most studies have indicated the important role of specific and non-specific reductases in the reduction of Se oxyanions, a few studies have demonstrated the role of flagellins in the mechanisms of interaction with heavy metals and other toxic compounds. Previous studies have suggested the possible role of flagellin-like proteins produced by the strain of our study, *S. bentonitica*, in the stabilization, synthesis, and transformation of SeNPs (Ruiz-Fresneda et al. 2018). Specifically, they seem to act as a template for SeNP growth and Se allotropic transformation. Our study provides the first evidence that flagellin-like proteins are strongly activated in *S. bentonitica* when toxic Se is added, supporting our previous hypothesis. Other studies have identified the role of flagellin protein FliC in controlling the assembly and stability of SeNPs in *R. aquatilis* HX2, but not their role in SeNP transformation (Li et al. 2021). More recently, flagellin FlaA produced by *Paenibacillus pabuli* AL109b has been shown to be directly involved in Te(IV) reduction (Farias et al. 2021). These results suggest that flagellin proteins, along with the previously mentioned thioredoxin, play a crucial role in the reduction and transformation to crystalline Se(0) NPs.

When the total membrane fraction in the presence of Se(VI) was supplemented with NADH (total membrane + +), the protein profile is not significantly altered. The proteins significantly affected in the total membrane $++$ treatment are detailed in Supplementary Table 3, Fig. 6, and Supplementary Fig. 5B. We observed a particular emphasis on the alterations of the Ax21 protein family. The Ax21 family protein showed a negative ratio of $-$ 3.85, indicating suppression compared to the control treatment (Supplementary Table 3; Fig. 6). Previous studies have shown that the loss of Ax21 through mutation leads to several phenotypic changes, including reduced motility, decreased biofilm formation, and lowered tolerance to toxic compounds, such as antibiotics (An and Tang 2018). Indeed, processes related to biofilm formation exhibited significant alteration in our samples (Supplementary Fig. 5B). These results indicate further evidence of the toxic effect exerted by Se(VI). Furthermore, Ax21 protein is believed to be released to the media within outer membrane vesicles (OMVs) and acting then as quorum-sensing factors (Ferrer-Navarro et al. 2013). The previously mentioned downregulation may also be indicative of a decreased release of these vesicles in an attempt to reduce the outer membrane permeability, as reported by Crossman et al. (2008). In their experiments, they demonstrated *S. maltophila* intrinsic resistance mechanisms include reduced outer membrane permeability, changes in LPS structure, and production of efflux pumps (Crossman et al. 2008). Alteration of the signalling and translocation processes detected in our samples support the previously mentioned suggestion (Supplementary Fig. 5B).

#### Statistical analysis of biological processes enrichment in the presence of Se(VI)

**Fig. 7:**
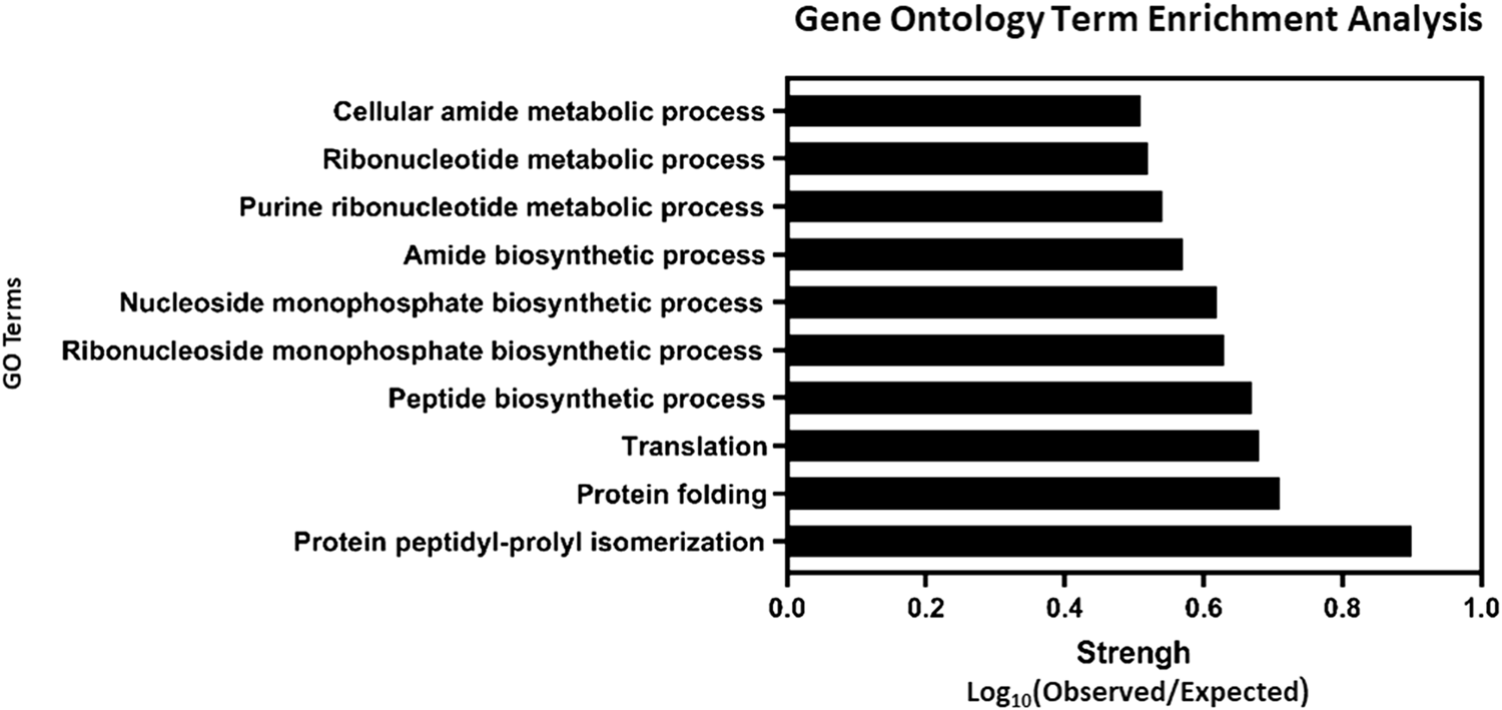
Gene Ontology (GO) term enrichment analysis of upregulated processes in the presence of Se(VI)

The results of the statistical analysis of biological process enrichment in the presence of Se(VI) were integrated into the Gene Ontology (GO) gene annotation system. Our primary focus was on scrutinizing the GO terms associated with proteins from *S. bentonitica* when it was exposed to Se(VI). The main objective was to analyse those general processes that are more activated in the presence of this stress-inducing agent. Amongst the 297 proteins subjected to analysis, STRING effectively provided annotations for 240 of them. These annotations uncovered 48 biological processes that exhibited significant enrichment and, therefore, were notably activated in the presence of Se(VI). The top 10 biological processes of greatest relevance in all treatments supplemented with Se(VI) (cytoplasm $++$ and $+-$; total membrane $++$ and $+-$) are depicted in Fig. 7. Amongst the metabolic processes that showed higher enrichment, we can highlight those linked to specific pathways responsible for the synthesis of proteins and nucleic acids. Specifically, a high activity of translation, isomerization, and the protein folding processes are worth noting, all of which are crucial during protein synthesis and regulation (Fig. 7). These results match very well with the differential emPAI ratios obtained previously, where chaperones, ribosomal proteins, and isomerases presented positive ratio values under Se(VI) stress (e.g. Chaperonin GroL, nucleoside monophosphate kinase, polyribonucleotide nucleotidyltransferase, peptidylprolyl isomerase) (Supplementary Table 2–3). Similarly, the enhancement of the metabolic pathways associated with the generation of ribonucleotides or nucleotide monophosphates (Fig. 7) was also identified to be upregulated (e.g. nucleoside-diphosphate kinase, 6-phosphogluconate dehydrogenase, polyribonucleotide nucleotidyltransferase, etc.) (Supplementary Table 2–3). These results demonstrate the strong adaptive response exerted to cope with such stressful conditions. Interestingly, the processes related to the synthesis and regulation of biomolecules appear to be more activated than those associated with energy acquisition, as they were previously detected to be downregulated for some proteins and upregulated for others.

## Conclusions

Our study demonstrates the ability of cytoplasmic and membrane sub-cellular fractions of the bacterium *S. bentonitica* to reduce toxic Se(VI) to Se(0)NPs in the presence and absence of NADH. A slowed structural and morphological transformation process from spherical and amorphous SeNPs to trigonal Se nanorods was only detected with electron microscopy in the total membrane fractions. It can be concluded that whilst complete cellular functionality is more effective, it is not essential for the reduction process, as protein cell fractions can carry this out. However, for the crystallization and transformation of the nanoparticles to be fully effective, the entire cellular machinery is required. The results evidence that the reduction of Se(VI) in *S. bentonitica* is a multi-modal process in which various mechanisms, at both cytoplasmic and membrane levels, are involved.

Proteomic studies revealed the disruption of several metabolic processes in the presence of Se(VI). Specifically, the perturbation of processes related to energy acquisition (succinate and malate dehydrogenase), regulation and remodelling of proteins and nucleic acids (chaperonin GroL, nucleoside diphosphate kinase, etc.), as well as membrane transport systems (TonB), reflect the adaptive responses exerted under this stress condition. results indicate the thioredoxin system as one of the main pathways involved in the reduction mechanism. Se reduction could also be conducted by Painter-type reactions most probably due to the accumulation of the glutathione precursor oxoglutarate through the truncation of the Krebs cycle and the upregulation of the thioredoxin system. Flagellin might also be involved in the Se allotropic transformation process observed in the reduced Se(0)NPs. Our results here reported could be of great help for the future development of new applications to be used in the bioremediation of Se-contaminated environments. Moreover, *S. bentonitica* emerges as a viable option for the recovery and synthesis of Se(0) NPs with a promising potential in diverse applications in both the industrial and biomedical sectors, aligning with the principles of a sustainable circular economy.

Supplementary information.

## Supplementary Information

Below is the link to the electronic supplementary material.

Supplementary file1 (DOCX 4236 KB)

## Data Availability

All supporting data generated and/or analyzed during this study are available from the corresponding author upon request.
