# Supplementary material for: Identification of a multi-modal mechanism for Se(VI) reduction and Se(0) allotropic transition by Stenotrophomonas bentonitica
Source: Environ Sci Pollut Res Int. 2024 Jul 12;32(28):16845–60. doi: 10.1007/s11356-024-34256-z (PMC12325488; doi:10.1007/s11356-024-34256-z)
Supplement: Supplementary file 1 — Supplementary file1 (DOCX 4236 KB) [file 11356_2024_34256_MOESM1_ESM.docx]

**Identification of a multi-modal mechanism for Se(VI) reduction and Se(0) allotropic transition by *Stenotrophomonas bentonitica***

Miguel Angel Ruiz-Fresneda^1,#,*^, Guillermo Lazúen-López^1,#^, Eduardo Pérez-Muelas^1^, Jesús Peña-Martín^2,3^, Raúl Eduardo Linares-Jiménez^1,†^, Antonio Martín Newman-Portela^1^, Mohamed Larbi Merroun^1^

^1^ Department of Microbiology, Campus Fuentenueva, University of Granada, 18071 Granada, Spain

^2^ Department of Human Anatomy and Embryology, Faculty of Medicine, University of Granada, 18016 Granada, Spain

^3^ Centre for Biomedical Research (CIBM), Biopathology and Regenerative Medicine Institute (IBIMER), University of Granada, 18100 Granada, Spain

^†^ Current address: Helmholtz-Zentrum Dresden-Rossendorf, Institute of Resource Ecology, Dresden, Germany

**^*^ Author for correspondence:** Miguel Angel Ruiz-Fresneda ([mafres@ugr.es](mailto:mafres@ugr.es))

**^#^** Miguel Angel Ruiz-Fresneda and Guillermo Lazuen-Lopez contributed equally

**Supplementary Figure 1**


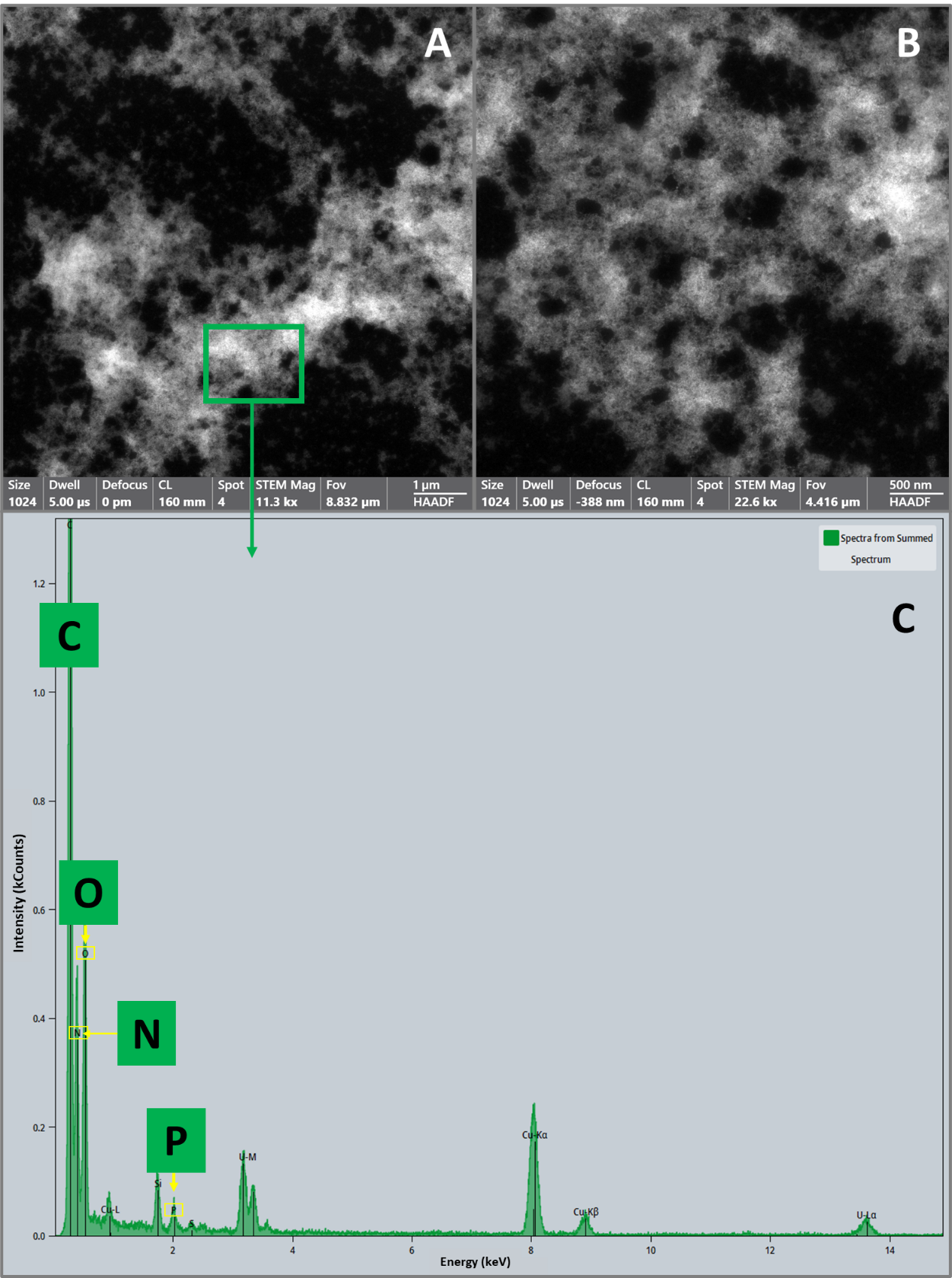


**Supplementary Figure 1.** TEM micrographs from the inner (A) and outer (B) membrane fractions of *S. bentonitica* supplemented with Se(VI) and NADH. A dense organic matrix mainly composed of nitrogen [N], oxygen [O], carbon [C], and phosphorus [P] as indicated by EDX analysis (C) and a complete absence of SeNPs could be observed.

**Supplementary Figure 2**


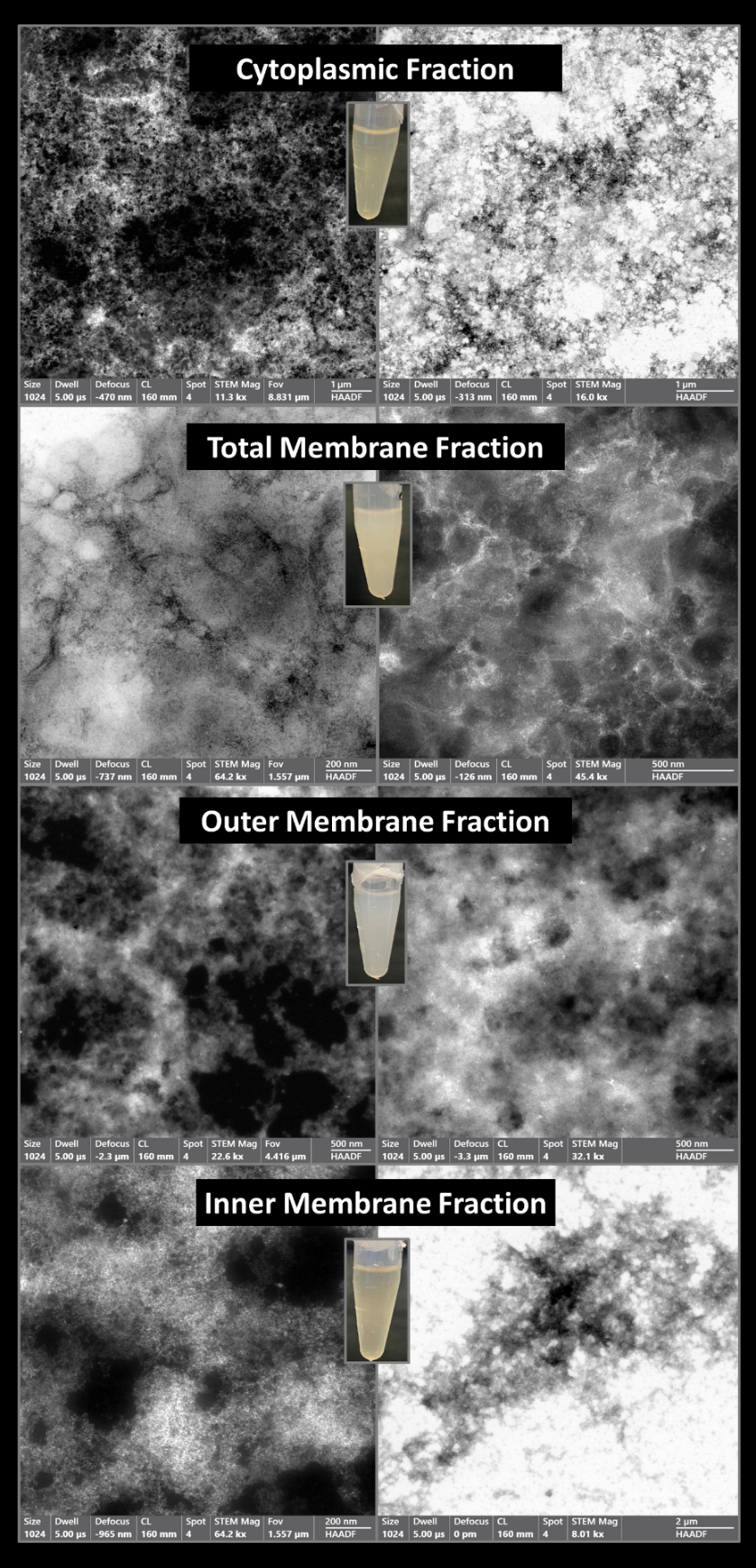


**Supplementary Figure 2.** TEM micrographs showing the absence of SeNPs in the different subcellular fractions (cytoplasmic, total-, outer-, and inner-membrane) controls without Se(VI).

**Supplementary Figure 3**

**
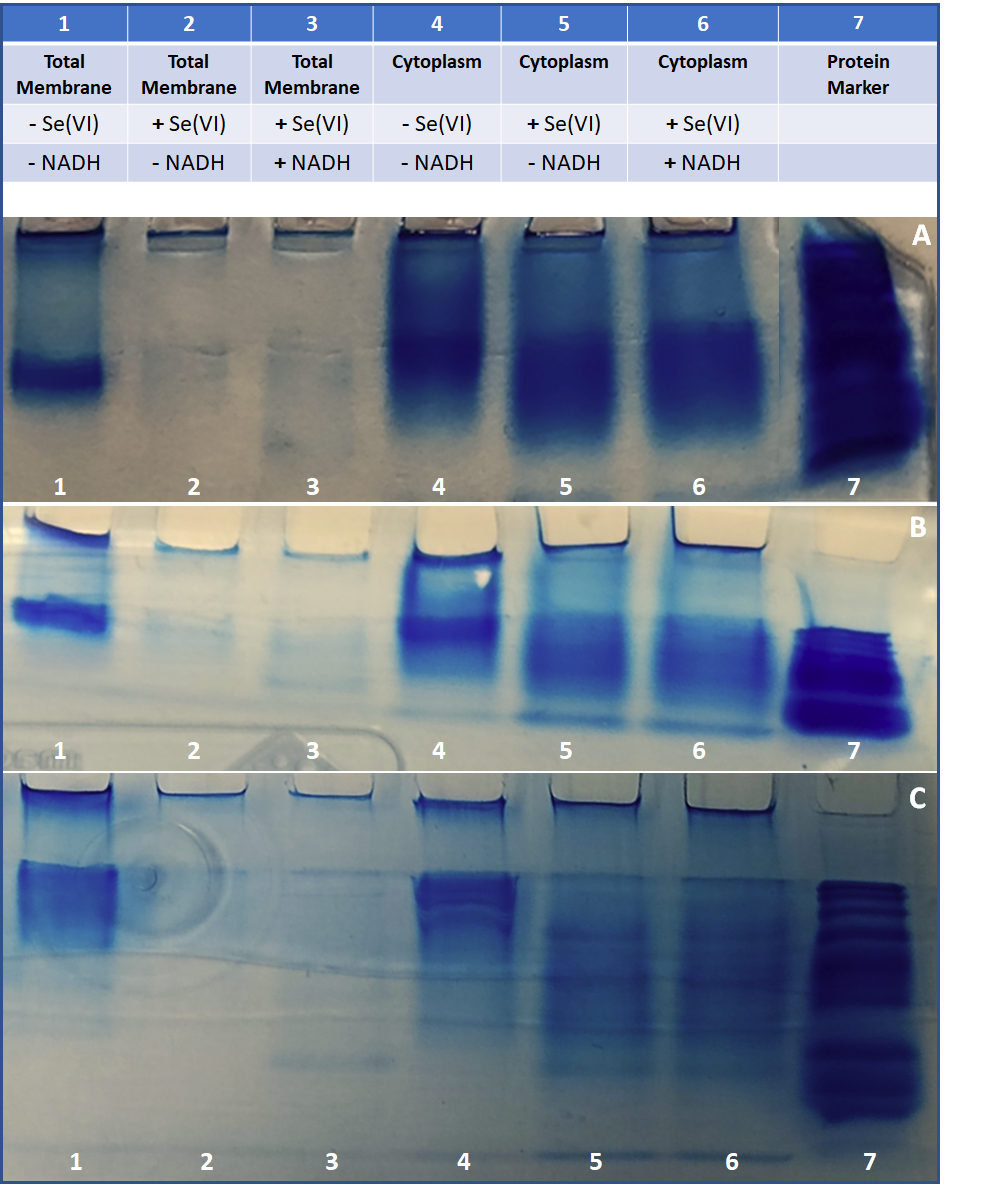
**

**Supplementary Figure 3.** SDS-PAGE analysis of different subcellular fractions (total membrane and cytoplasm) of the bacterium *S. bentonitica* in the presence and absence of both Se(VI) and NADH. The analysis was performed in triplicates (A: replicate 1; B: replicate 2, and C: replicate 3).

**Supplementary Figure 4**

**
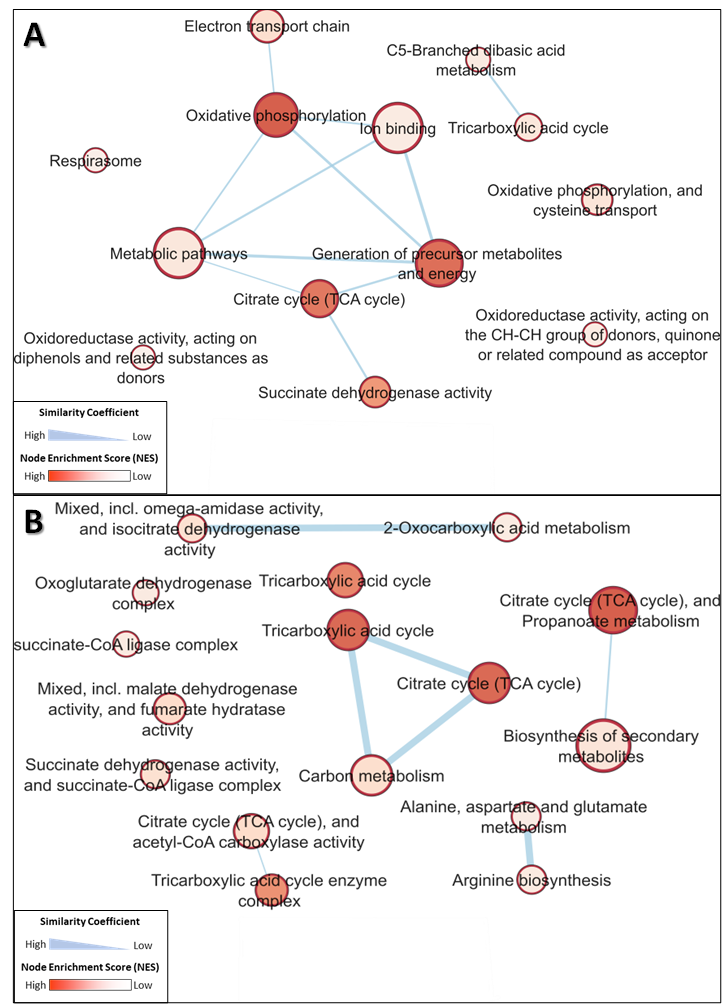
**

**Figure S4.** Metabolic pathway enrichment network within the cytoplasmic compartment supplemented with A) Se(VI) (Cytoplasm $+-$) and B) Se(VI) and NADH (Cytoplasm $++$) in comparison to the control in the absence of Se(VI) (Cytoplasm $--$). Graphic legend: The node size is associated with the number of altered proteins belonging to that process; The node colour indicates the NES, which measures the intensity of the alteration and the thickness of the connections indicates the similarity coefficient between the connecting nodes (measured by the quantity of proteins these nodes shared)

**Supplementary Figure 5**

**
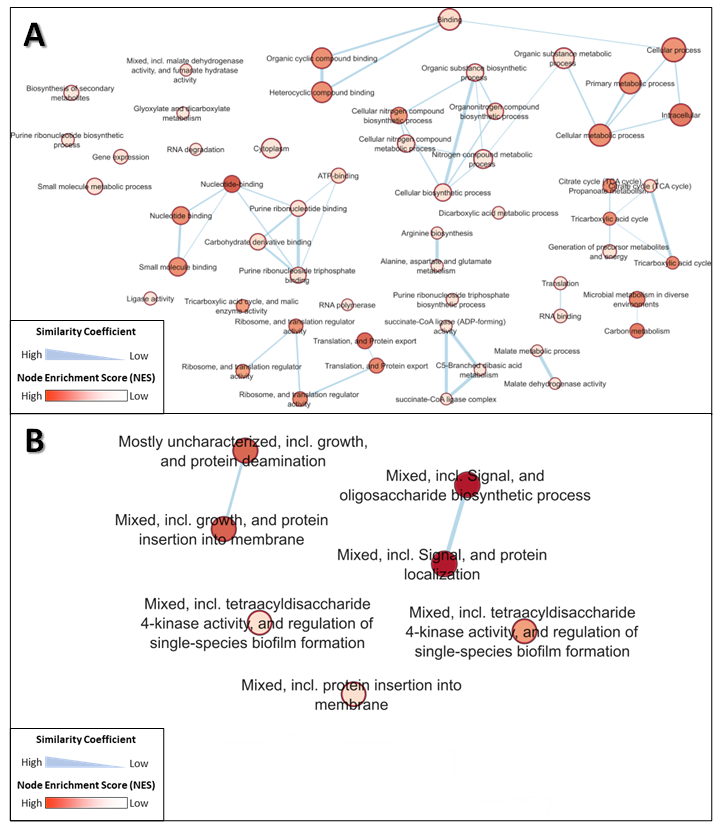
Figure S5.** Metabolic pathway enrichment network within the Total membrane compartment supplemented with A) Se(VI) (Total membrane $+-$) and B) Se(VI) and NADH (Total membrane $++$) in comparison to the control in the absence of Se(VI) (Total membrane $--$). Graphic legend: The node size is associated with the number of altered proteins belonging to that process; The node colour indicates the NES, which measures the intensity of the alteration and the thickness of the connections indicates the similarity coefficient between the connecting nodes (measured by the quantity of proteins these nodes shared).

**Supplementary Table 1**

**Supplementary Table 1.** Summary of the nomenclature for the different treatments and controls used in the enzymatic reduction assays. Note: (++) represents NADH (10mM) and Se(VI) 200mM are supplemented; (+-) represents Se(VI) is supplemented; and (- -) represents the absence of both NADH and Se(VI).

| **Subcellular fraction** | **Treatment** | **Sample name** |
| --- | --- | --- |
| **Cytoplasm** | NADH (10mM); Se(VI) 200mM ; Tris-HCl (10mM pH 7.5); 0.5 mg/ml protein extract | **Cytoplasm** $\boldsymbol{++}$ |
|  | Se(VI) 200mM ; Tris-HCl (10mM pH 7.5); 0.5 mg/ml protein extract | **Cytoplasm** $\boldsymbol{+-}$ |
|  | Tris-HCl (10mM pH 7.5); 0.5 mg/ml proteín extract | **Cytoplasm** $\boldsymbol{--}$ |
| **Total membrane** | NADH (10mM); Se(VI) 200mM ; Tris-HCl (10mM pH 7.5); 0.5 mg/ml protein extract | **Total membrane** $\boldsymbol{++}$ |
|  | Se(VI) 200mM ; Tris-HCl (10mM pH 7.5); 0.5 mg/ml protein extract | **Total membrane** $\boldsymbol{+-}$ |
|  | Tris-HCl (10mM pH 7.5); 0.5 mg/ml protein extract | **Total membrane** $\boldsymbol{--}$ |
| **Inner membrane** | NADH (10mM); Se(VI) 200mM ; Tris-HCl (10mM pH 7.5); 0.5 mg/ml protein extract | **Inner membrane** $\boldsymbol{++}$ |
|  | Se(VI) 200mM ; Tris-HCl (10mM pH 7.5); 0.5 mg/ml protein extract | **Inner membrane** $\boldsymbol{+-}$ |
|  | Tris-HCl (10mM pH 7.5); 0.5 mg/ml protein extract | **Inner membrane** $\boldsymbol{--}$ |
| **Outer membrane** | NADH (10mM); Se(VI) 200mM; Tris-HCl (10mM pH 7.5); 0.5 mg/ml protein extract | **Outer membrane** $\boldsymbol{++}$ |
|  | Se(VI) 200mM ; Tris-HCl (10mM pH 7.5); 0.5 mg/ml protein extract | **Outer membrane** $\boldsymbol{+-}$ |
|  | Tris-HCl (10mM pH 7.5); 0.5 mg/ml protein extract | **Outer membrane** $\boldsymbol{--}$ |
| **Positive control** | NADH (10mM); Se(VI) 200mM; *S. bentonitica* BII-R7 culture (10%); LB | **Positive control** $\boldsymbol{++}$ |
|  | Se(VI) 200mM; *S. bentonitica* BII-R7 culture (10%); LB | **Positive control** $\boldsymbol{+-}$ |
|  | *S. bentonitica* BII-R7 culture (10%); LB | **Positive control** $\boldsymbol{--}$ |
| **Negative control** | NADH (10mM); Se(VI) 200mM; Tris-HCl (10mM pH 7.5) | **Negative control** $\boldsymbol{++}$ |
|  | Se(VI) 200mM; Tris-HCl (10mM pH 7.5) | **Negative control** $\boldsymbol{+-}$ |
|  | Tris-HCl (10mM pH 7.5) | **Negative control** $\boldsymbol{--}$ |

**Supplementary Table 2**

**Supplementary Table 2**. Proteins from the cytoplasmic fraction of *S. bentonitica* in the presence of Se(VI) (Cytoplasm +-) and in the presence of Se(VI) and NADH (Cytoplasm ++) which have significantly altered ratios compared to the negative control (Cytoplasm - -).

| **Accesion ID** | **Gene name** | **Description** | **p. adj.** | **Cytoplasm** $\boldsymbol{+-}$**/**$\boldsymbol{--}$ **Ratio** |
| --- | --- | --- | --- | --- |
| OEY99525.1 | BIY45_16325 | Succinate dehydrogenase iron-sulfur subunit [Stenotrophomonas sp. BIIR7] | 0.000797 | -4.55 |
| OEY99684.1 | BIY45_15500 | Peptidase [Stenotrophomonas sp. BIIR7] | 0.0373 | -3.92 |
| **Accesion ID** | **Gene name** | **Description** | **p. adj.** | **Cytoplasm** $\boldsymbol{++}$**/**$\boldsymbol{--}$ **Ratio** |
| OEY99011.1 | BIY45_19190 | Hypothetical protein BIY45_19190 [Stenotrophomonas sp. BIIR7] | 0.0206 | -4.06 |
| OEY99056.1 | BIY45_18920 | Hypothetical protein BIY45_18920 [Stenotrophomonas sp. BIIR7] | 0.0281 | -2.55 |
| OEY99525.1 | BIY45_16325 | Succinate dehydrogenase iron-sulfur subunit [Stenotrophomonas sp. BIIR7] | 0.00345 | -3.21 |
| OEY99537.1 | BIY45_16385 | Hypothetical protein BIY45_16385 [Stenotrophomonas sp. BIIR7] | 0.0129 | -3.42 |
| OEY99655.1 | BIY45_15700 | DNA-binding response regulator [Stenotrophomonas sp. BIIR7] | 0.02 | 2.78 |
| OEY99700.1 | BIY45_15425 | Serine-type D-Ala-D-Ala carboxypeptidase [Stenotrophomonas sp. BIIR7] | 0.0238 | -3.53 |
| OEY99797.1 | BIY45_14935 | Aminopeptidase [Stenotrophomonas sp. BIIR7] | 0.000221 | -4.28 |
| OEY99817.1 | BIY45_14780 | Efflux transporter periplasmic adaptor subunit [Stenotrophomonas sp. BIIR7] | 0.0422 | -3.78 |
| OEY99841.1 | BIY45_14740 | Hybrid sensor histidine kinase/response regulator [Stenotrophomonas sp. BIIR7] | 0.0085 | -3.36 |
| OEY99936.1 | BIY45_14250 | Dipeptidyl carboxypeptidase II [Stenotrophomonas sp. BIIR7] | 0.0334 | -2.78 |
| OEZ00035.1 | BIY45_13760 | Hypothetical protein BIY45_13760 [Stenotrophomonas sp. BIIR7] | 0.0168 | -3.01 |
| OEZ00141.1 | BIY45_13190 | Hypothetical protein BIY45_13190 [Stenotrophomonas sp. BIIR7] | 0.0305 | -3.29 |
| OEZ00143.1 | BIY45_13200 | Isocitrate dehydrogenase (NADP(+)) [Stenotrophomonas sp. BIIR7] | 0.0332 | 2.64 |
| OEZ00213.1 | BIY45_12745 | Thiol:disulfide interchange protein [Stenotrophomonas sp. BIIR7] | 0.0343 | -3.34 |
| OEZ00725.1 | BIY45_10305 | Peptidylprolyl isomerase [Stenotrophomonas sp. BIIR7] | 0.045 | -3.72 |
| OEZ01063.1 | BIY45_08330 | Peptidase [Stenotrophomonas sp. BIIR7] | 0.00324 | -4.2 |
| OEZ01255.1 | BIY45_07425 | Competence protein [Stenotrophomonas sp. BIIR7] | 0.0173 | -2.82 |
| OEZ01381.1 | BIY45_06550 | Thioredoxin [Stenotrophomonas sp. BIIR7] | 0.0341 | 3.17 |
| OEZ01840.1 | BIY45_04155 | Spermidine/putrescine ABC transporter substrate-binding protein PotF [Stenotrophomonas sp. BIIR7] | 0.0259 | -3.54 |
| OEZ01866.1 | BIY45_04320 | Oar protein [Stenotrophomonas sp. BIIR7] | 0.0101 | -3.82 |
| OEZ01929.1 | BIY45_04060 | Hypothetical protein BIY45_04060 [Stenotrophomonas sp. BIIR7] | 5.13E-05 | -6.2 |
| OEZ01972.1 | BIY45_03740 | Polyketide cyclase [Stenotrophomonas sp. BIIR7] | 2.16E-05 | -5.73 |

**Supplementary Table 3**

**Supplementary Table 3**. Proteins from the total membrane fraction of *S. bentonitica* in the presence of Se(VI) (Total membrane +-) and in the presence of Se(VI) and NADH (Total membrane ++) which have significantly altered compared to the negative control (Total membrane - -).

| **Accesion ID** | **Gene name** | **Description** | **p. adj.** | **Total membrane** $\boldsymbol{+-}$**/** $\boldsymbol{--}$ **Ratio** |
| --- | --- | --- | --- | --- |
| OEY99537.1 | BIY45_16385 | Hypothetical protein BIY45_16385 [Stenotrophomonas sp. BIIR7] | 0.0143 | 3.44 |
| OEY99609.1 | BIY45_15915 | 30S ribosomal protein S8 [Stenotrophomonas sp. BIIR7] | 0.00474 | 3.29 |
| OEY99706.1 | BIY45_15465 | 6-phosphogluconate dehydrogenase (decarboxylating) [Stenotrophomonas sp. BIIR7] | 0.036 | 2.71 |
| OEZ00531.1 | BIY45_11205 | TonB-dependent receptor [Stenotrophomonas sp. BIIR7] | 0.0131 | -3.27 |
| OEZ00859.1 | BIY45_09330 | Polyribonucleotide nucleotidyltransferase [Stenotrophomonas sp. BIIR7] | 0.0326 | 3.05 |
| OEZ01113.1 | BIY45_08235 | Chaperonin GroL [Stenotrophomonas sp. BIIR7] | 0.000104 | 5.72 |
| OEZ01186.1 | BIY45_07830 | Malate dehydrogenase [Stenotrophomonas sp. BIIR7] | 0.0109 | 4.01 |
| OEZ01359.1 | BIY45_06805 | Citrate (Si)-synthase [Stenotrophomonas sp. BIIR7] | 0.031 | 3 |
| OEZ01592.1 | BIY45_05780 | Nucleoside-diphosphate kinase [Stenotrophomonas sp. BIIR7] | 0.00891 | 3.53 |
| WP_171966697.1 |  | Flagellin [Stenotrophomonas bentonitica] | 0.0062 | 3.3 |
| WP_171966698.1 |  | Flagellin [Stenotrophomonas bentonitica] | 0.0249 | 3.32 |
| **Accesion ID** | **Gene name** | **Description** | **p. adj.** | **Total membrane** $\boldsymbol{++}$**/** $\boldsymbol{--}$ **Ratio** |
| OEY99537.1 | BIY45_16385 | Hypothetical protein BIY45_16385 [Stenotrophomonas sp. BIIR7] | 0.000216 | 3.81 |
| OEZ00058.1 | BIY45_13465 | Ax21 family protein [Stenotrophomonas sp. BIIR7] | 0.00026 | -3.8 |
| OEZ00602.1 | BIY45_10610 | Biopolymer transporter ExbB [Stenotrophomonas sp. BIIR7] | 0.0313 | 2.85 |
